# Riots and subways, a relationship moderated by the neighborhood’s income level

**DOI:** 10.1038/s41598-022-14859-7

**Published:** 2022-06-22

**Authors:** Carlos Cartes, Kenzo Asahi, Rodrigo Fernández

**Affiliations:** 1 Complex Systems Group, Facultad de Ingeniería y Ciencias Aplicadas, Universidad de Los Andes, Santiago, Chile; 2 Escuela de Gobierno, Pontificia Universidad Católica de Chile, Santiago, Chile; 3 Centre for Sustainable Urban Development (CEDEUS), Santiago, Chile; 4 Millennium Nucleus on Intergenerational Mobility: From Modelling to Policy (MOVI), Santiago, Chile; 5 Facultad de Ingeniería y Ciencias Aplicadas, Universidad de Los Andes, Santiago, Chile

**Keywords:** Psychology and behaviour, Statistics

## Abstract

Social disturbances due to socioeconomic and political factors received media attention during 2019 in places like France, Hong Kong, Chile, Nigeria, Sudan, Haiti, and Lebanon. In October 2019, Chile saw massive demonstrations in the capital city of Santiago. The cost of damage to infrastructure during the first month of unrest was estimated at US$ 4.6 billion, and the cost to the Chilean economy was about US$ 3 billion, 1.1% of its Gross Domestic Product. This study analyzes how the topology of the public transport network affected the locations of the 2019 riots in Santiago. On average, we find a clear association between proximity to the subway network and riot density. This association is significant only in neighborhoods with residents in the highest and lowest income quartiles. As a result, when analyzing social unrest and the critical role of public transport, policymakers should also consider the crucial role of income.

## Introduction

In October 2019, Chile saw massive demonstrations in the capital city of Santiago, triggered by a 3.8% rise in Metro fares. However, the increase in the subway fare was not the reason for the protests, but they were due to the quality of the health, education, and pension systems, among other demands. The slogan of the demonstrations was: "This is not for the 30 pesos, but the last 30 years [since the return to democracy]" (30 Chilean pesos is equivalent to 4 US cents). Within a few days, protesters turned violent, and the social turmoil rapidly spread to other cities, forcing the Government to declare a state of emergency and impose military controls. As stated by Hribernik and Haynes^1^, the cost of damage to infrastructure during the first month of unrest was estimated at US$ 4.6 billion, and the cost to the Chilean economy was about US$ 3 billion, 1.1% of its Gross Domestic Product.

The public disorder had a multifactorial origin. Some authors state that economic hardship is a relevant factor in predicting riot severity and duration. Pioneering studies pointed out that poverty, coming from ethnic segregation, explained riots in the USA during the 1960s^2–4^. Later research investigating the London 2011 riots found that deprivation and poor economic conditions were the main factors influencing which areas of the city were more prone to violence in case of disorder. In addition, there is some indication that criminal opportunism does not entirely explain the riots' spatial distribution^5,6^, thus hypothesizing that we should consider other psychological mechanisms. Hence, in Santiago, a metropolis with inequalities^7^, we hypothesize that the association between subway proximity and riot intensity is heterogeneous across the neighborhoods’ income levels. In the case of the Paris riots of 2005, the primary sociodemographic variable that may have led to the revolts was the proportion of young males aged between 16 and 24 with incomplete high school education^8^. More recent studies, for the case of the French riots, found that regular criminal activity was not affected by the disorder activity of 2005^9^. In ‘Gilets Jaunes’ incidents in French cities during 2018, Boyer et al.^10^ found an association between online and rioting activity.

Meanwhile, Pires and Crooks^11^ pointed out that employment opportunities decrease social tension. They use an agent-based model, social network analysis, and geographical information. They conclude that if only education is increased, the result is a more tense and unstable social context due to the rise of frustration and civil unrest. Therefore, we hypothesize that educational covariates are relevant, as they could affect riot intensity and be correlated with proximity to the subway network.

Quantitative studies related to the disorders in Latin America are scarce. Cartes and Davies developed a numerical model that reproduces the basic features of Santiago's riots by implementing a simple Latin American city pattern^12^. We can also point to an analysis that uses diffusion–reaction equations, applied to country-wide economic variables, and predict civil unrest due to population dissatisfaction^13^.

This article analyzes how the topology of Santiago’s subway network is associated with the 2019 riots. To our knowledge, we provide the first quantitative study on the relationship between riot density, the urban transport network, and the neighborhood’s income level. Previous evidence from the London riots in 2011^14^ shows that transport network hubs enable riot formation. Also, past research^15,16^ shows that segregation is associated with social tension and violence. Moreover, an appreciable number of studies^17–19^ indicate a clear correlation between increased accessibility, allowed by an improved transport network, and criminal activity because of more significant exposure to potential offenders or new occasions for those likely offenders.

Based on the previously cited research, we test how distance from the transport network correlates with riot density. Also, we extend previous work by exploring heterogeneities in this association across income levels. We found a significant, negative association between distance to the subway network and riot density, albeit only substantial in Santiago’s highest and lowest income neighborhoods (quartiles one and four, respectively).

## Methods

### Accessibility

We present a measure of accessibility to determine how easy it is to move within a city using its public transport network. Similar metrics have been proposed before^20–22^; however, we aim to define a flexible and general formulation that can be applied to any network. This formulation allows us to understand how accessibility is spatially distributed in a city and how this can be affected by changes to the transport network. The concept of *extended network* is introduced based on the area around each network node (i.e., a subway station) accessible to the users. This area is related to the distance people are willing to walk to use public transport.

The proportion of this extended network accessible to a user by taking only one means of transport, given the geographic position, is what we call *accessibility*. This measure is associated with the concept of potential accessibility^23^ as it depends on the location of the network nodes and how they are interconnected. The result is a bi-dimensional distribution showing the accessibility inside a city.

To implement the accessibility measure, we assumed a network composed of ${n}_{b}$ lines. The location of each station in the city and its expanded line will be stored on independent arrays of size ${n}_{x}\times {n}_{y}$. The array stores the subway and expanded networks, called $b$ and $e$, respectively. Each element belonging to those arrays will have a value of either $0$ or $1$. If the line $k$ ($k=1,\dots ,{n}_{b}$) has a station at the position $(i,j)$ then the value of the array element belonging to the line at that position will have a value of $1$ ($0$ otherwise). This is written as ${b}_{i,j}^{(k)}=1$. Associated with this subway line, the expanded network is defined in the following way: if ${b}_{i,j}^{(k)}=1$ then the elements of the expanded network related to the $k$ line ${e}^{(k)}$ will have the values of $1$ at the position $(i,j)$ and its surroundings. Therefore, it follows that for the $k$ line and $(i,j)$ position,

1 $${e}_{i,j}^{(k)}={e}_{i\pm 1,j}^{(k)}={e}_{i,j\pm 1}^{(k)}={e}_{i\pm 1,j\pm 1}^{(k)}=1$$

The square-shape pattern generated by this expanded network was used because of its simplicity and ease of implementation.

Using the previous definitions, the entire expanded network can be written as follows:

2 $$R_{{i,j}} = P\left[ {\sum\limits_{{k = 1}}^{{n_{b} }} {e_{{i,j}}^{{(k)}} } } \right]$$

where $P$ is an operator acting on each element of the array ${\sum }_{k=1}^{{n}_{b}}{e}_{i,j}^{(k)}$, with the following definition:

3 $$P\left[a\right]=\left\{\begin{array}{c}1 if a>0\\ 0 if a \le 0\end{array}\right.$$

This operator is used to normalize the size of each element in ${R}_{i,j}$ and avoid double count at places where the expanded networks overlap. With the use of Eq. (2), we can define the total expanded network size as the sum of all its elements in the following way:

4 $$N_{t} = \sum\limits_{{i = 1}}^{{n_{x} }} {\sum\limits_{{j = 1}}^{{n_{y} }} {R_{{i,j}} } }$$

Now the accessible region of the plane for each particular position given by the pair $(i,j)$ can be defined as the array:

5 $$f_{{l,m}}^{{(i,j)}} = P\left[ {\sum\limits_{{k = 1}}^{{n_{b} }} {e_{{i,j}}^{{(k)}} e_{{l,m}}^{{(k)}} } } \right]$$

The product of the $e$ arrays implies that the expanded lines considered have non–zero elements at $(i,j)$ to avoid multiple counts at the overlapping points.

Finally, we can calculate the numerical value of the accessibility for the position $(i,j)$ as the fraction of the total expanded network that can be reached from a specific location:

6 $$A^{{(i,j)}} = \frac{1}{{N_{t} }}\sum\limits_{{l = 1}}^{{n_{x} }} {\sum\limits_{{m = 1}}^{{n_{y} }} {f_{{l,m}}^{{(i,j)}} } }$$

### Empirical specification

We use regression techniques to explore the heterogeneity in the association between proximity to the subway network and riot intensity across the neighborhoods’ income levels. Other methods, such as propensity score matching, are less prone to describe associations when the key variable (in this case, the distance to the subway network) is continuous and are also less prone to heterogeneity analyses. We measure the distance between the coordinates of each incident and its closest subway station as the spherical distance among them (as the crow flies).

We describe a bivariate relationship between distance to the subway network and riots in the following way,

7 $${Y}_{i}={\beta }_{0}+{\beta }_{1}{d}_{i}+{\varepsilon }_{i}$$

where ${Y}_{i}$ is the natural logarithm of the number of riots reported in grid cell $i$, ${d}_{i}$ is the distance between the grid cell’s centroid and the closest subway station (our key variable), and ${\varepsilon }_{i}$ is the error term.

Because of potential omitted variables that could be biasing a careful association between subway proximity and riot intensity, we include covariates such as the neighborhood’s income level and educational variables, dimensions identified in previous research as determinants of riots^8,24^. Hence, the following equation depicts the empirical specification including covariates:

8 $${Y}_{i}={\beta }_{0}+{\beta }_{1}{d}_{i}+{{\varvec{i}}{\varvec{n}}{\varvec{c}}}_{{\varvec{i}}}^{\boldsymbol{^{\prime}}}{{\varvec{\beta}}}_{2}+{{\varvec{x}}}_{{\varvec{i}}}^{\boldsymbol{^{\prime}}}{{\varvec{\beta}}}_{4}+{\varepsilon }_{i}$$

in this case, ${{\varvec{i}}{\varvec{n}}{\varvec{c}}}_{{\varvec{i}}}^{\boldsymbol{^{\prime}}}$ is a vector with dummies for quartiles of the neighborhood’s average income level and ${{\varvec{x}}}_{{\varvec{i}}}^{\boldsymbol{^{\prime}}}$ is a vector of educational covariates.

Because we are interested in exploring the heterogeneity in the association between distance to the subway network and distance to the subway network, we include the interaction between distance to the subway network and neighborhood’s income level:

9 $${Y}_{i}={\beta }_{0}+{\beta }_{1}{d}_{i}+{{\varvec{i}}{\varvec{n}}{\varvec{c}}}_{{\varvec{i}}}^{\boldsymbol{^{\prime}}}{{\varvec{\beta}}}_{2}+{d}_{i}\times {{\varvec{i}}{\varvec{n}}{\varvec{c}}}_{{\varvec{i}}}^{\boldsymbol{^{\prime}}}{{\varvec{\beta}}}_{3}+{{\varvec{x}}}_{{\varvec{i}}}^{\boldsymbol{^{\prime}}}{{\varvec{\beta}}}_{4}+{\varepsilon }_{i}$$

in Eq. (9), the vector ${{\varvec{\beta}}}_{3}$ captures the degree of heterogeneity in the association between distance to the subway network and riot density across income quartiles.

## Results

**Figure 1:**
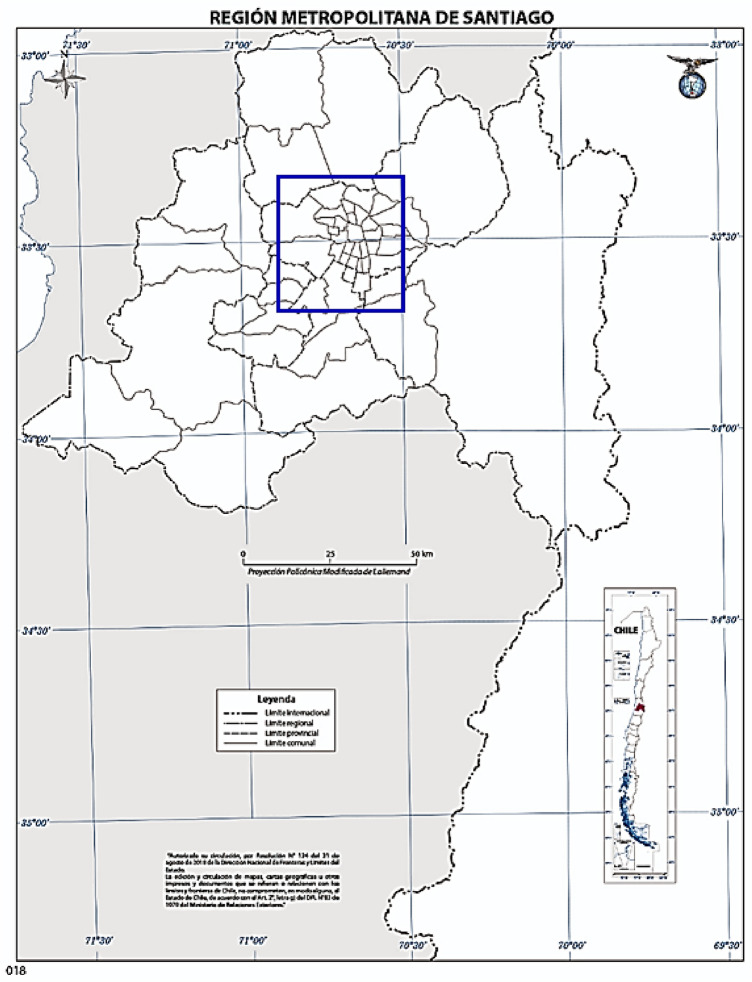
Study area of the riots (blue square). Bottom right: location of the Santiago Metropolitan Region in the Chilean territory (in purple). https://www.curriculumnacional.cl/portal/Educacion-General/Historia-Geografia-y-Ciencias-Sociales-1-basico/HI01-OA-09/132560:Region-Metropolitana-mudo [free resources from the Ministry of Education, Chile].

We took the public data from SOSAFE (sosafeapp.com), an open platform on which users can report various incidents. These incidents may be blockades that disrupt road traffic, destroy street signs, loot supermarkets and stores, or arson public and private premises. Data were taken for the first four days of demonstrations (October 18th–22nd, 2019). We performed a space–time analysis as the data included georeferenced and timestamped incidents. This analysis's area was the same as that covered by Santiago’s public transport system, including the entire bus and subway networks. We considered all data belonging to the region delimited by the parallels − 33° 19′ and − 33° 40′ latitude South and the meridians 70° 29′ and 70° 52′ longitude West. They correspond to a total of 5002 reported events. The raw data is publicly available. Figure 1 shows a map of Santiago, alongside its position in Chile.

### Riots’ geographic characteristics

#### Temporal evolution

**Figure 2:**
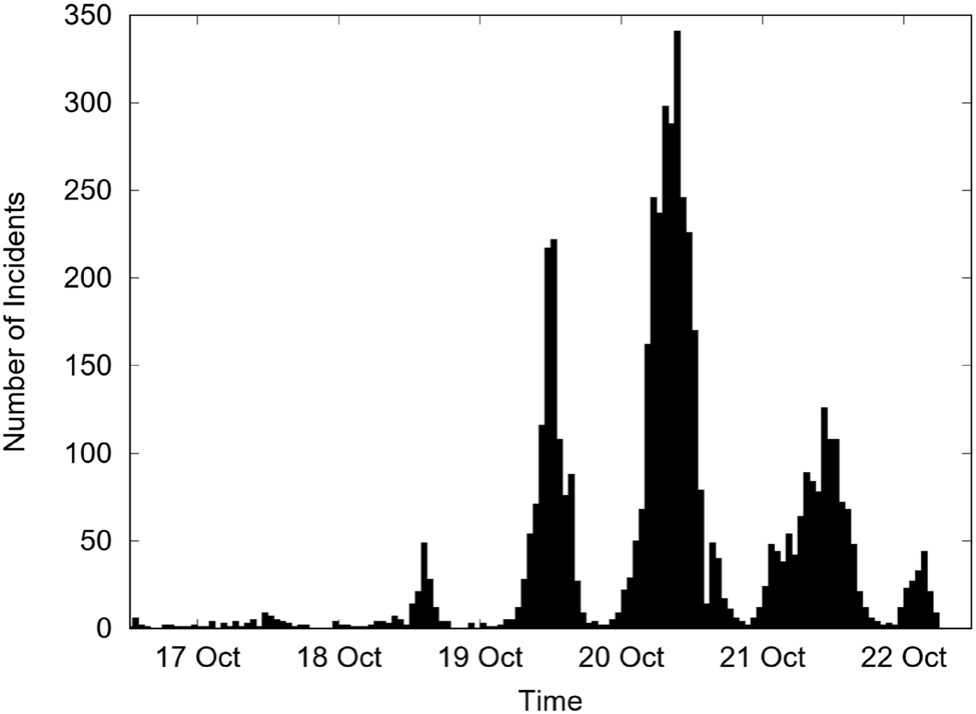
Number of incidents per hour for the first four days of rioting.

Figure 2 shows the temporal evolution of public disorder incidents, where each bar represents one hour of activity. Figure 2 shows that the activity is lowest around 8 a.m. and peaks at 10 p.m. This kind of dynamics, where the number of rioting events exhibits rapid growth followed by exponential decay, was first described by Burbeck et al.^3^. This temporal behavior was also observed during the French riots of 2005^8^ and the London riots of 2011^24^. In recent work, Caroca Soto et al.^25^, using country-wide data collected by the Undersecretary of Human Rights for the Chilean Riots of 2019, confirmed Burbeck’s model.

#### Spatial distribution relative to the subway network

**Figure 3:**
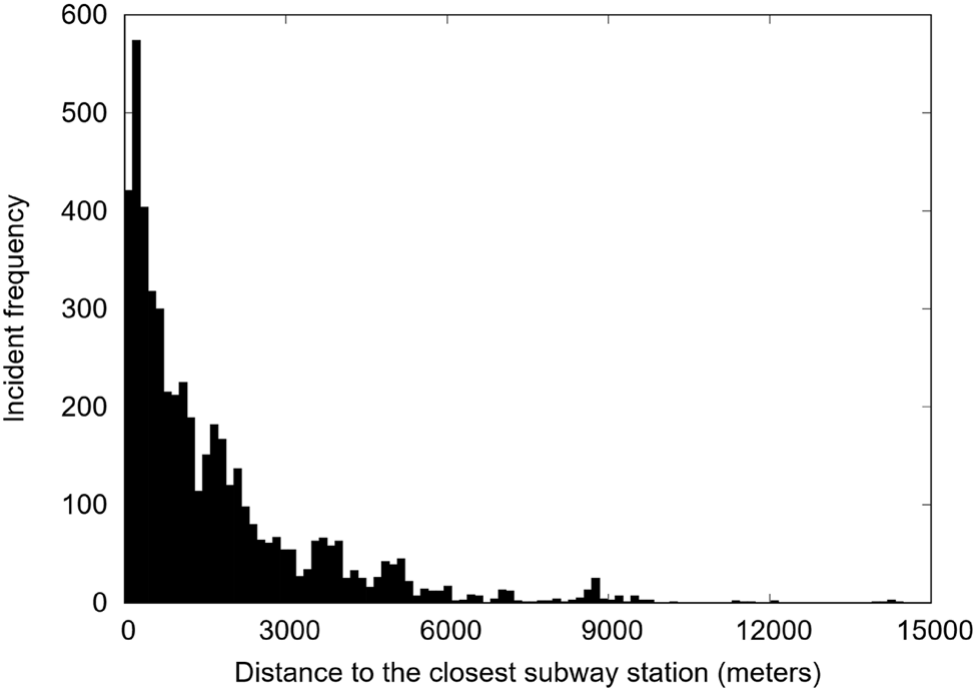
Frequency distribution of distances between incidents and subway stations.

Figure 3 shows the frequency of incidents against the distance to the closest subway station. This is measured as the spherical distance between the reported incident and the nearest subway station. There is an exponential decay in the activity as we move further away from the stations: 48.7% of all incidents occur one km or less from a station, and 82.8% occur three km or less. This activity clustering around the subway network was the same on each day of social disorder.

**Figure 4:**
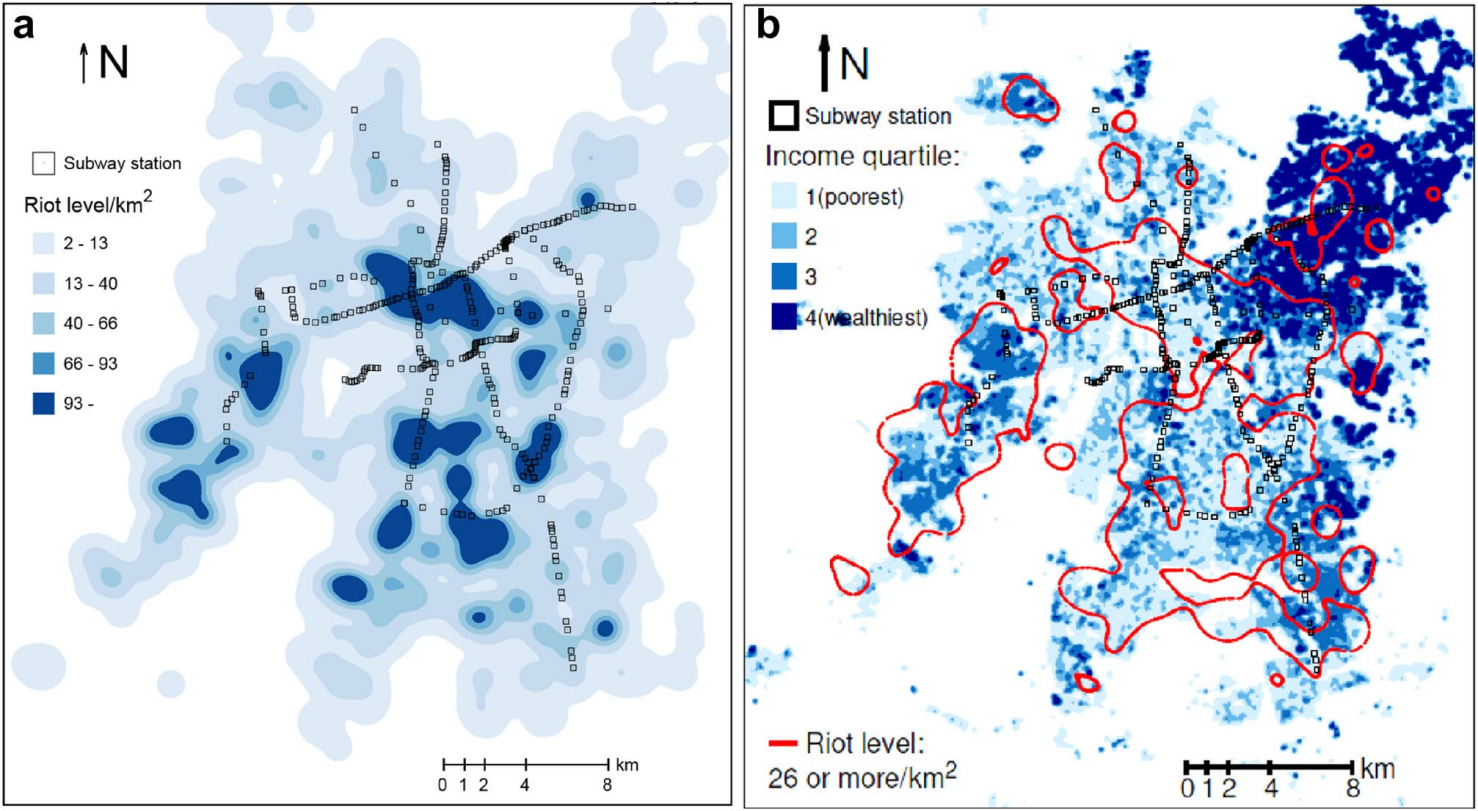
Incident density, the subway network, and income distribution.

Using the measure of accessibility presented in Eq. (6) (see “Methods”), we found that the subway network covers only 12.4% of the total area served by the public transport system Santiago with 7 lines, 140 km, and 136 stations. The rest of the system is made by the bus system with 6600 vehicles, 370 routes, 2800 km, and 10,000 bus stops. Figure 4a shows a heat map of riots alongside the subway network, where the rioting level is measured as the number of reported events by km^2^. There is a visible association between the location of subway stations and riot density because most of the regions registering a high density of events, with 93 or more reported incidents by km^2^, are close or share the position with the subway network. The previous visual association is substantial in high-income neighborhoods. See the city’s northeast in Fig. 4b, where the inhabitants’ income is categorized by quartiles, ranging from the poorest (quartile 1) to the wealthiest (quartile 4). This income distribution is shown alongside the surface’s boundaries, where the reported incidents are 26 or more per km^2^ (red lines),where the rioting level is measured as the number of reported events by km^2^. There is a visible association between the location of subway stations and riot density because most of the regions registering a high density of events, with 93 or more reported incidents by km^2^, are close or share the position with the subway network. The previous visual association is substantial in high-income neighborhoods. See the city’s northeast in Fig. 4b, where the inhabitants’ income is categorized by quartiles, ranging from the poorest (quartile 1) to the wealthiest (quartile 4). This income distribution is shown alongside the surface’s boundaries, where the reported incidents are 26 or more per km^2^ (red lines).

**Figure 5:**
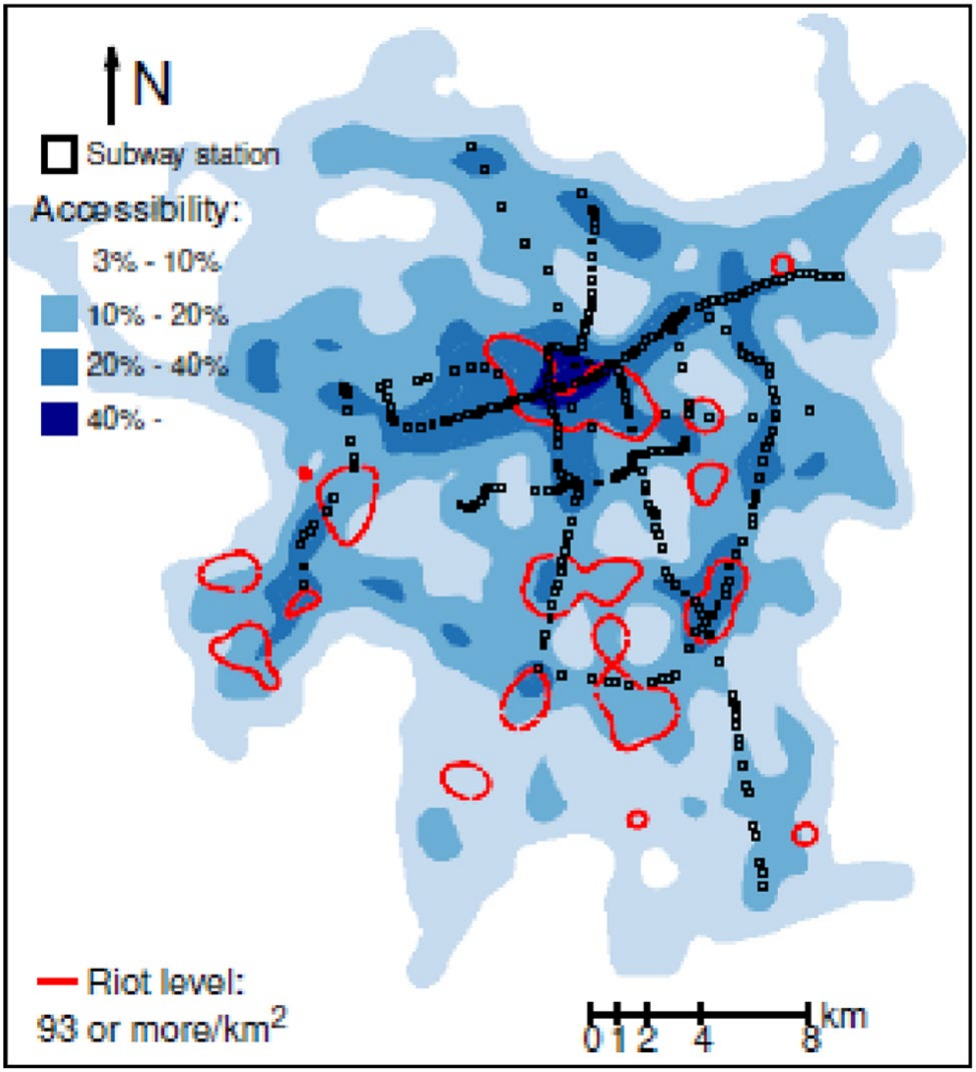
Accessibility heat map and contour lines of riot intensity.

Figure 5 shows a heatmap of the spatial distribution of accessibility, computed using Eq. (6), alongside the subway network and contour lines for the regions of highest rioting intensity, with 93 or more events per km^2^. Almost all these high activity regions lay close to areas reachable from 20% or more of Santiago’s total surface.

### Regression analysis of riots

#### The association between proximity to the subway network and riot density

There is a strong negative association between distance to the subway network and riot density. Table 1 shows regression coefficients and standard errors of a one-kilometer increment change in distance from the subway network. In this analysis, the dependent variable is the log of riots per grid cell, where cells have a width and height of 200 m. Our results were consistent when performing the same analysis with cells ranging from 100 to 500 m. Column (1) shows that every kilometer from the subway network is associated with a 4.4% decrease in riots (a coefficient of − 4.327). Column (2) shows that this association is robust to including income and educational covariates (a coefficient of − 3.734).

**Table 1:** The Association Between Distance from the Subway Network and Riots Across Income Levels.

Dependent variable: log (riots)	(1) Bivariate regression	(2) As (1) plus covariates	(3) As (2) interacting proximity with income
Distance to the subway network (km)	− 4.327*** (1.323)	− 3.734*** (1.355)	− 11.39*** (3.072)
First income quartile		0.246 (5.317)	− 3.774 (8.549)
Second income quartile		3.455 (4.968)	− 11.72 (7.954)
Third income quartile		10.24** (4.913)	− 1.752 (7.848)
Fourth income quartile (reference category)		0 (0)	0 (0)
**Distance to the subway network × **			
First income quartile			4.804 (3.948)
Second income quartile			11.68*** (3.837)
Third income quartile			10.31** (4.045)
Fourth income quartile (reference category)			0 (0)
Free schools’ value-added	No	Yes	Yes
Paid schools’ value-added	No	Yes	Yes
Observations	1,879	1,879	1,879
R-squared	0.006	0.015	0.020

Columns (1) through (3) follow Eqs. (7) through (9), respectively. The table reports regression coefficients and standard errors multiplied by 100 to give the percentage effect of a one-km change in distance. Columns (2) and (3) control for nearby publicly-funded and privately-funded school value-added in each cell, where gaussian weights decrease with school distance. In column (1), we restricted the sample to our preferred specification’s (column 3) sample to increase comparability across columns. We divided the Greater Santiago Area into a grid of 200 × 200 m. We restrict the sample to those areas closer than five kilometers from the closest subway station. We calculated schools’ value-added as the coefficient on a dummy on each school type. In these regressions to determine each school’s value-added, the dependent variable is standardized test scores, and the covariates are parental education and income. Robust standard errors are in parentheses. All regressions include an intercept (not shown). ***p < 0.01, **p < 0.05, *p < 0.1.

#### A heterogeneous association across income levels

**Figure 6:**
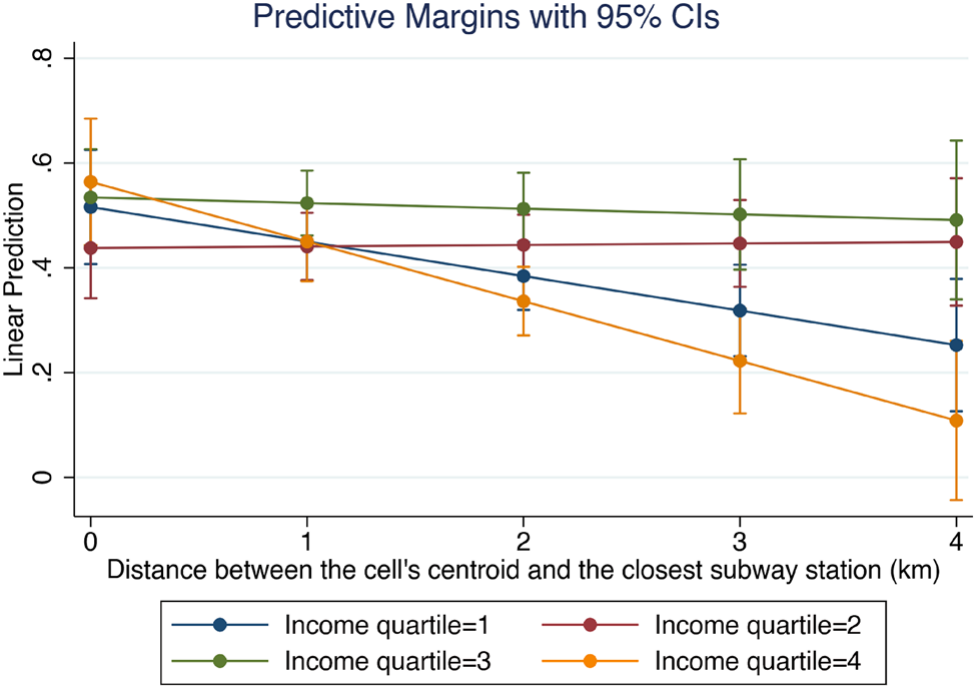
The marginal association between distance to the subway network and riot density across neighborhood income levels. The first and fourth income quartiles are the poorest and wealthiest, respectively. This figure displays the results in Table 1, column (3).

Both Santiago’s wealthiest and most impoverished areas drive the association between proximity to the subway network and riot density. Column (3) shows that the association between distance to the subway network and riot density is more substantial in neighborhoods with predominantly poorest or wealthiest residents (first and fourth income quartiles). Figure 6 shows the marginal association between distance to the subway network and riot density for neighborhoods of different income levels. As can be seen in the figure, the negative slope is steeper (in absolute terms) in the wealthiest (fourth income quartile) and poorest (first income quartile) neighborhoods. The mentioned slope in areas with residents closer to median income (neighborhoods with an average income in the second and third quartiles) is small in statistical and practical terms.

In other words, the further away the station is, the lower the number of riots, but only in the case of the poorest and wealthiest areas of the city, which is not the case in middle-income neighborhoods where the distance to the stations has no influence.

## Discussion

We presented a simple way to quantify the association between riot density and access to the subway network allowing for heterogeneity across neighborhoods’ income levels. Spatial and temporal data distributions from Chile’s 2019 riots indicated that the riots peaked around 10 p.m. Half of the riots took place one kilometer or less from subway stations, showing an exponential decay with distance.

The association between riot density and proximity to the subway network is significant, both statistically and in policy terms. For example, a neighborhood five kilometers closer to the subway network has a riot density 22% higher than a neighborhood farther away. This is of particular importance for the city of Santiago, as mobility studies indicate that most inhabitants tend to either stay in their community or travel to the city center^26^. Therefore, it is not strange that the city center has the highest concentration of rioting activity. Unfortunately, we do not have data to test whether most rioters traveled long distances to participate in city center demonstrations. However, anecdotal evidence suggests that at least a fraction of the rioters traveled from the suburbs to riot^27^.

In this work, our goal was to explain the association between proximity to the subway network and riot density, not to predict it. Therefore, we focus more on the key variables’ statistical significance rather than on the regressions’ R^2^. However, the R^2^ still has a role. Low R2 as in the analyses in Table 1 implies that there are relevant explanatory variables that are not in our model. However, for omitted variable bias to arise, we need an omitted variable that is correlated with the outcome variable and the analyzed key variable. Future work could delve into the role of variables distinct to income and education that could affect riot density and the proximity to the subway network.

The association between proximity to the subway network and riot density is significant only in the lowest- and highest-income neighborhoods. We hypothesize that the reason for the previously mentioned association in areas in the lowest income quartile is that inequality (one of the riots’ motivations, according to Somma et al.^28^) burdens low-income families^29^. The association between distance from the subway network and rioting activity in high-income neighborhoods is remarkable because it has not been reported in previous work. The area of rioting activity is concentrated around the subway network. One explanation is that rioters in high-income areas come from low-income neighborhoods in the city’s Northwest and use the subway network for their journey to riot. By contrast, rioters in low-income communities of the city’s south and, to a lesser extent, the southwest live nearby and are less likely to use the subway network on their way to the riot. This can be seen by overlaying Fig. 4a,b. When analyzing social unrest and the critical role of public transport, policymakers should consider the crucial role of income in the previous relationship.
